# Lesion location and serum levels of homocysteine are associated with early‐onset post‐stroke depression in acute ischemic stroke

**DOI:** 10.1002/brb3.3210

**Published:** 2023-08-16

**Authors:** Hongxu Zhou, Chenlong Wang, Wei Wang, Hongyan Li, Qun Hu, Ni Huang, Yining Huang

**Affiliations:** ^1^ Department of Neurology Civil Aviation General Hospital of Peking University Beijing China; ^2^ Department of Neurology Peking University First Hospital Beijing China

**Keywords:** acute ischemic stroke, early onset, homocysteine, lesion location, post‐stroke depression

## Abstract

**Introduction:**

It is well known that post‐stroke depression (PSD) is a psychiatric complication after stroke which leads to worse functional outcome and poorer quality of life. Some risk factors including gender, stroke severity, lesion location, homocysteine (HCY), and so on are associated with PSD. This study aims to further explore the possible relationship between serum levels of HCY and early‐onset PSD and the predictive value of HCY combined with stroke characteristics for early‐onset PSD.

**Methods:**

Two hundred forty‐five patients with acute ischemic stroke who met the criteria were included in this study from March 2015 to March 2017. PSD was diagnosed at 2 weeks after stroke. The severity of depressive symptoms was evaluated with the Hamilton depression scale 17 items (HAMD‐17), and patients with HAMD scores ≥7 were included in the PSD group. The demographic data, clinical characteristics, serum levels of HCY, and detailed radiological variables (e.g., lesion location and quantity of the brain infarct) were also examined.

**Results:**

In total, 97 (39.6%) patients of the 245 patients were diagnosed with depression. The univariate analyses suggested that patients in PSD group had a higher NIHSS score, modified Rankin Scale score, and HCY levels than patients in non‐PSD group (*p <* .001). The patients with PSD had higher proportion of multiple‐site acute infarcts and frontal lobe lesion (*p <* .05). In multivariate logistic regression analysis, NIHSS score at admission, serum levels of HCY, and multiple‐site lesions were independently related to early‐onset PSD. Based on receiver operating characteristic curves analysis, the combination of HCY, NIHSS scores, multiple‐site lesions, and lesion location revealed a highest area under the curve of 0.807 (95% confidence interval [CI]: 0.748–0.865, *p <* .001). Furthermore, there was a significantly increased risk of early‐onset PSD associated with serum levels of HCY ≥16.98 μmol/L (odds ratio [OR] = 10.976, 95% CI: 5.585–21.573, *p <* .001).

**Conclusions:**

Our study indicated that higher NIHSS score, elevated serum levels of HCY, and multiple‐site lesions may be independent risk factors of early‐onset PSD. The combination of HCY, NIHSS scores, multiple‐site lesions, and lesion location may provide greater predictive value than HCY alone for early‐onset PSD. Early intervention for elevated serum levels of HCY may be a potential target for the intervention and prevention of PSD.

## Graphical abstract

Our study indicated that higher NIHSS score, elevated serum HCY levels and multiple‐site lesions may be independent risk factors of early‐onset PSD. The combination of HCY, NIHSS scores, multiple‐site lesions and lesion location may provide greater predictive value than HCY alone for PSD. Early intervention for elevated serum HCY levels may be a potential target for prevention of PSD.

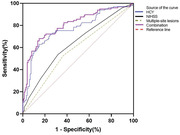

## 1 INTRODUCTION

Post stroke depression (PSD) is a severe and frequent complication of mental disorders followed by stroke. The clinical manifestations of PSD are characterized by low mood and loss of interest, accompanied by slow thinking, sleep disorder, sense of worthless, sense of guilt, and even suicidal tendency (Wang et al., 2016). PSD is one of the multiple important factors that reduces the quality of life and neurological rehabilitation of patients. The prevalence rate of PSD is about 33% within 1 month after stroke. Depression reduces the desire of active rehabilitation of patients after stroke, negatively affects the recovery of neurological function and cognitive function of patients, and increases the mortality rate (Cai et al., 2019; Wang et al., 2016). Many studies have already attempted to investigate the pathophysiological mechanism of PSD, but there is still a lack of universal explanation on the mechanism of PSD. Researchers have found that many risk factors are associated with PSD, such as age, gender, history of depression, stroke severity, lesion localization, and inflammatory cytokines (Guo et al., 2022). Homocysteine (HCY) is an essential intermediate product during the metabolism of methionine to cysteine. Many studies showed that HCY is an independent risk factor for cardiovascular and cerebrovascular diseases, especially for ischemic stroke (Han et al., 2015; Poddar, 2021; Yuan et al., 2021). Elevated HCY can damage vascular endothelial cells, affect vasodilation function, promote vascular smooth muscle proliferation, and participate in inflammatory reaction and oxidative stress leading to the occurrence and development of atherosclerosis and thrombosis (Larsson et al., 2019; Poddar, 2021; T. Zhang et al., 2020). For these reasons, hyperhomocysteinemia is also considered to be highly associated with ischemic stroke. Meanwhile, some studies have indicated that reducing HCY levels by supplementing vitamin B including vitamin B6, B12, and folic acid can reduce the risk of ischemic stroke (X. Huang et al., 2017; Kataria et al., 2021; Li et al., 2016). Previous studies have reported that elevated serum levels of HCY at admission were associated with PSD at 3 months to 1 year after stroke (Cheng et al., 2018; Li et al., 2017; Pascoe et al., 2012). Other studies have reported that the combination of high level HCY and high sensitivity C‐reactive protein (CRP) can more effectively predict early stage PSD (Cheng et al., 2018; Yin et al., 2018). However, there have not been comprehensive studies on the composite effect of HCY, stroke severity, and stroke lesion localization on PSD in the past. This study explored the relationship between serum HCY levels and early‐onset PSD by detecting serum HCY levels of patients at 2 weeks after acute ischemic stroke. Meanwhile, we evaluated both the association between different brain lesion locations and PSD and the association between the combination of HCY, stroke severity, and lesion localization and PSD. Our study's objective was to find clues that these factors will increase prognostic information in the early evaluation of PSD.

## 2 MATERIALS AND METHODS

### 2.1 Study population

Patients with acute ischemic stroke who were hospitalized at the Department of Neurology of Civil Aviation General Hospital during the period from March 2015 to March 2017 were included in this study. This study was approved by the ethics committee of Civil Aviation General Hospital (IRB approval number: 2023‐L‐K‐14). Written informed consent was obtained from all study participants or their immediate family members before participation. The inclusion criteria were as follows: (1) patients who met the diagnostic criteria for ischemic stroke (the 4th Chinese Academic Conference for Cerebrovascular Diseases), acute stroke was verified from computed tomography (CT) or magnetic resonance imaging (MRI) within 24 h after admission in all patients, (2) patients aged between 18 and 85 years old, and (3) patients admitted to the hospital within the first 72 h after stroke onset were consecutively recruited. The exclusive criteria were as follows: (1) severe aphasia or dysarthria and consciousness disorder to unable to complete evaluations and questionnaires; (2) pre‐stroke diagnosis of dementia or significant cognitive impairment; (3) combined with severe heart or liver or renal insufficiency; (4) self‐report of having any psychiatric illness including depression and/or using psychotropic drugs prior to stroke onset; (5) medical histories of other central nervous system diseases, for example, Parkinson's disease and epilepsy; and (6) malignant tumor and nutritional disorders which will probably cause metabolic abnormalities.

### 2.2 Clinical variables and neuroimaging

At the baseline, clinical information was collected at admission, including demographic data (age and sex), history of conventional vascular risk factors (hypertension, diabetes mellitus, coronary heart disease, hyperlipidemia, and a family history of stroke), and clinical characteristics. Smoking history, alcohol consumption, and prior or acute treatment were obtained. Smoking was defined as a patient who had smoked continuously for 5 years with at least 10 cigarettes per day. Alcohol consumption was defined as a patient who had drunk continuously for 5 years with at least 20 g ethanol per day. The National Institutes of Health Stroke Scale (NIHSS) was used to evaluate stroke severity at admission by trained neurologists (Brott et al., 1989). NIHSS scores were assessed within 24 h after admission. Moderate‐to‐severe stroke was defined as an NIHSS score ≥5. The Barthel Index (BI) scores were assessed at discharge. In addition, the functional outcome was also assessed according to the modified Rankin Scale (mRS) score at follow‐up after 1 month. An mRS score ≥3 indicated an unfavorable functional outcome (Chi et al., 2021).

Relevant tests such as electrocardiography, blood pressure, blood routine test, and biochemical test were carried out for all participants. Systolic blood pressure and diastolic blood pressure were measured within the first 4 h after admission. Mean arterial pressure was calculated and recorded. MRI were performed within 24 h after admission to assess the site, cause, and quantity of the brain infarct. MRI was performed using a stroke protocol, including T1‐, T2‐, and diffusion‐weighted imaging sequences.

### 2.3 Clinical assessment and subject grouping

According to Diagnostic and Statistical Manual of Mental Disorders, 5th edition (DSM‐V) (American Psychiatric Association, 2013), trained neurologists and psychiatrists diagnosed patients with PSD at 2 weeks after stroke onset. The severity of depressive symptoms was evaluated with the Hamilton depression scale 17 items (HAMD‐17) (Hamilton, 1960). According to the recommendation, HAMD‐17 scores <7 mean normal condition (Y. Zhang et al., 2017), and patients with these scores were enrolled in the non‐PSD group. Patients with HAMD‐17 scores greater than or equal to 7 were included in the PSD group. A score of 7−17, 18−23, and more than 24 indicates mild depression, moderate depression, and severe depression, respectively (Shen et al., 2019; Y. Zhang et al., 2017; Zimmerman et al., 2013). According to the scores obtained, patients were classified into the mild, moderate, or severe PSD groups.

### 2.4 Blood sample collection and laboratory test

Blood samples were collected via venipuncture in ethylenediaminetetraacetic acid (EDTA) BD Vacutainer tubes at 6:00 a.m. after admission. Blood samples were centrifuged by 3000 rpm for 10 min, and the upper serum was packed with an EP tube 15 min later and was stored in a −80°C refrigerator until the time of assay. The concentration of serum HCY was measured using the kit (Baiding Bioengineering Co., Ltd.) according to the manufacturer's instruction in our hospital's clinical laboratory. Fasting blood glucose, glycosylated hemoglobin (HbA1c), total cholesterol (TC), triglyceride (TG), low‐density lipoprotein (LDL), high‐density lipoprotein (HDL), and uric acid levels were also measured using standard laboratory methods by an automatic biochemical analyzer (Beckman AU5800).

### 2.5 Statistical analysis

The results of the categorical variables were expressed as percentages. Continuous variables were shown as the mean ± SD or the median and interquartile range (IQR) depending on the normal or non‐normal distribution of variables. Continuous variables were compared by Student's *t*‐test for normally distributed variables, while the Mann–Whitney *U* test was employed for non‐normally distributed variables. The comparison between proportions was made using Fisher's exact test or Chi‐square test. An univariable analysis was employed to compare the baseline and clinical differences between patients with PSD and without PSD. The influence of HCY or lesion location on PSD was determined by binary logistic regression analysis. The results were presented with adjusted odds ratios (ORs) and the corresponding 95% confidence intervals (CIs). Spearman's Rank correlation analysis was employed for bivariate correlations. Receiver operating characteristic curves (ROC) were used to test the accuracy of HCY, NIHSS, and lesion location as diagnostic tools to predict PSD. All statistical tests were performed with SPSS version 20.0 for Windows (IBM). All statistical assessments were two tailed, and *p* < .05 was considered to be statistically significant in all tests.

## 3 RESULTS

### 3.1 Baseline characteristics

**FIGURE 1:**
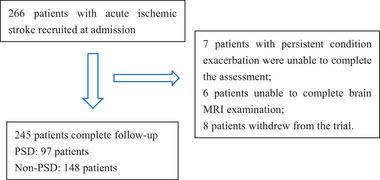
Study recruitment profile. PSD, post‐stroke depression.

The study cohort consisted of 266 patients with acute ischemic stroke at baseline. By the time of follow‐up at 2 weeks, there were 245 patients in our study, and 21 (7.9%) patients dropped out of the study (Figure 1). In those patients, the median age was 62 (IQR, 55–71) years and 163 (66.5%) were male. There were 32 (13.1%) patients with moderate‐to‐severe stroke (NIHSS ≥ 5) at admission with a median NIHSS score of 2 (IQR 1−3). The BI score at discharge was 85 (IQR 65−100). An unfavorable outcome (mRS score ≥ 3) at 1 month was found in 79 patients. In total, 97 of the 245 patients were diagnosed with depression for a percentage of 39.6%. The baseline characteristics of the patients in the two groups are shown in Table 1. Compared with the non‐PSD group, the PSD group showed a higher NIHSS score (3 [1–4] vs. 2 [1–3], *p <* .001), mRS score (2 [2–3] vs. 1 [1–2]; *p <* .001), HAMD score (14 [11–18] vs. 5 [4–6], *p <* .001), and lower BI score at discharge (75 [60–95] vs. 90 [75–100], *p <* .001). Compared with the non‐PSD group, patients with PSD had higher proportion of moderate‐to‐severe stroke (NIHSS score ≥ 5) (19 [19.6%] vs. 13 [8.8%], *p =*.014). The percentage of unfavorable outcome in the PSD group was much higher than that in the non‐PSD group (43 [44.3%] vs. 36 [24.3%], *p =*.001) at the 1‐month follow‐up. There were no significant differences between the PSD and non‐PSD groups in age, gender, history of vascular risk factors (hypertension, diabetes mellitus, coronary heart disease, hyperlipidemia, ischemic stroke, hemorrhagic stroke, and a family history of stroke), personal history (smoking history and alcohol consumption), and serum biochemical index (fasting blood glucose, HbA1c, TC, TG, LDL, HDL, uric acid, and CRP).

**TABLE 1:** Baseline characteristics of patients in the non‐post‐stroke depression (PSD) and PSD groups.

Characteristics	Non‐PSD (*n* = 148)	PSD (*n* = 97)	*p*‐Value
Demographic			
Age(years), median (IQR)	62.5 (56–71)	62 (55–73)	.757
Male, *n* (%)	100 (67.6)	63 (64.9)	.671
History of vascular risk factors, *n* (%)			
Hypertension	107(72.3)	71(73.2)	.877
Diabetes mellitus	52(35.1)	35(36.1)	.880
Coronary heart disease	29(19.6)	16(16.5)	.540
Hyperlipidemia	90(60.8)	57(58.8)	.749
Ischemic stroke	45(30.4)	31(32.0)	.797
Hemorrhagic stroke	4(2.7)	4(4.1)	.716
Family history of stroke	9(6.1)	13(13.4)	.050
Current cigarette smoking	83(56.1)	55(56.7)	.924
Current alcohol drinking	56(37.8)	36(37.1)	.909
Vital sign and biochemical index			
Systolic blood pressure, median (IQR), mmHg	155 (142–170)	160 (148–171)	.131
Diastolic blood pressure, median (IQR), mmHg	89 (80–95)	88 (79–100)	.677
TG, mean (SD), mmol/L	1.80 ± 1.17	2.01 ± 1.37	.194
TC, mean (SD), mmol/L	4.72 ± 1.04	4.89 ± 1.22	.239
HDL‐C, mean (SD), mmol/L	1.27 ± 0.30	1.27 ± 0.29	.890
LDL‐C, mean (SD), mmol/L	2.57 ± 0.80	2.73 ± 0.93	.142
FBG, median (IQR), mmol/L	6.07 (5.38–7.99)	5.96 (5.17–7.71)	.319
HbA1c, median (IQR), (%)	6.2 (5.8–7.8)	6.4 (5.7–7.2)	.685
Uric acid, mean (SD), μmol/L	341.94 ± 91.63	349.79 ± 101.82	.531
HCY, median (IQR), μmol/L	13.18 (10.44–15.72)	18.90 (14.38–23.48)	<.001
CRP, median (IQR), mg/L	1.74 (0.96–4.31)	2.50 (1.04–6.39)	.110
Neuropsychological evaluation			
NIHSS score, median (IQR)	2 (1–3)	3 (1–4)	<.001
Moderate to severe stroke (NIHSS ≥ 5)	13 (8.8)	19 (19.6)	.014
BI score, median (IQR)	90 (75–100)	75 (60–95)	.001
mRS score, median (IQR)	1 (1–2)	2 (2–3)	<.001
Unfavorable outcome (mRS ≥ 3)	36 (24.3)	43 (44.3)	.001
HAMD‐17 score, median (IQR)	5 (4–6)	14 (11–18)	<.001

Abbreviations: BI, Barthel Index; CRP, C‐reactive protein; FBG, fasting blood glucose; HAMD‐17, Hamilton depression scale 17 items; HbA1c, glycosylated hemoglobin; HCY, homocysteine; HDL‐C, high‐density lipoprotein cholesterol; IQR, interquartile range; LDL‐C, low‐density lipoprotein cholesterol; mRS, modified Rankin Scale; NIHSS, National Institutes of Health Stroke Scale; SD, standard deviation; TC, total cholesterol; TG, triglyceride.

### 3.2 Stroke lesion and PSD

The relationship between stroke lesion location and PSD in the two groups is shown in Table 2. Compared with non‐PSD group patients, patients with PSD had higher proportion of multiple‐site acute infarcts (51 [52.6%] vs. 54 [36.5%], *p =*.013). There are differences between the two groups in the specific location of acute infarction. Compared with patients without PSD , patients with PSD had higher proportion of Frontal lobe lesion (17 [17.5%] vs. 10 [6.8%], *p =*.008), lower proportion of Parietal lobe lesion (2 [2.1%] vs. 12 [8.1%], *p =*.046). There were no significant differences between the two groups in the Lateralization of acute infarction (*p >* .05).

**TABLE 2:** Relationship between stroke lesion location and post‐stroke depression (PSD).

Stroke lesion characteristics	Non‐PSD (*n* = 148)	PSD (*n* = 97)	*p*‐Value
Lateralization, *n* (%)			
Left hemisphere	53 (35.8)	30 (30.9)	.692
Right hemisphere	43 (29.1)	32 (33.0)	
Bilateral hemisphere	7 (4.7)	8 (8.2)	
Brain stem	31 (20.9)	20 (20.6)	
Cerebellum	14 (9.5)	7 (7.2)	
Quantity of acute infarct, *n* (%)			
Single	94(63.5)	46(47.4)	.013
Multiple	54(36.5)	51(52.6)	
Lesion location, *n* (%)			
Frontal lobe	10 (6.8)	17 (17.5)	.008
Temporal lobe	5 (3.4)	4 (4.1)	.743
Parietal lobe	12 (8.1)	2 (2.1)	.046
Occipital lobe	5 (3.4)	2 (2.1)	.707
Basal ganglia	61 (41.2)	40 (41.2)	.997
Thalamus	10 (6.8)	5 (5.2)	.609
Brain stem	31 (20.9)	20 (20.6)	.951
Cerebellum	14 (9.5)	7 (7.2)	.540

### 3.3 Serum levels of HCY and PSD

**FIGURE 2:**
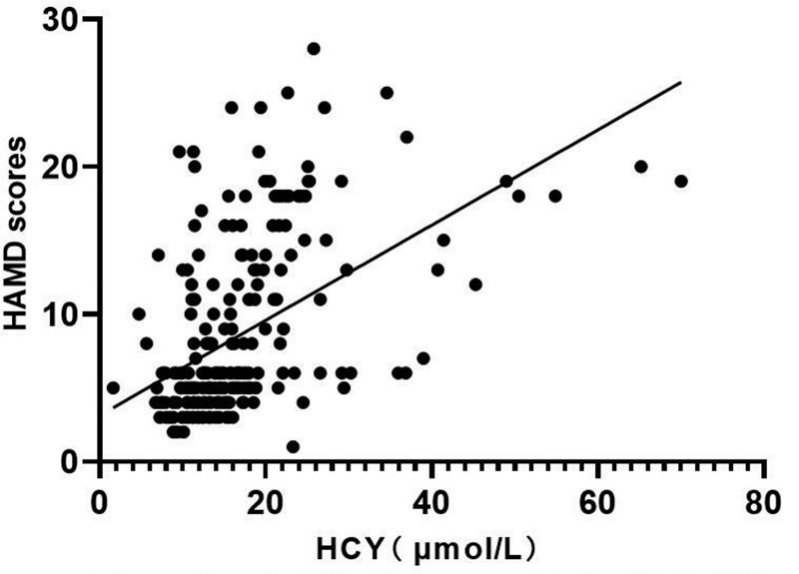
The correlation between serum levels of homocysteine (HCY) and the Hamilton depression scale (HAMD) scores in all patients (*n* = 245, *r* = 0.532, *p <* .001).

**FIGURE 3:**
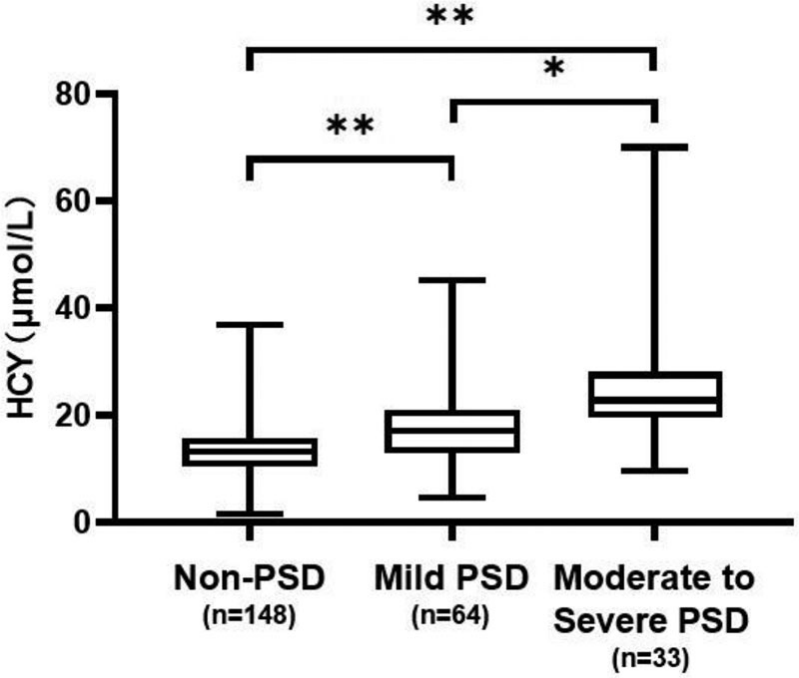
Comparison of serum homocysteine (HCY) level among post‐stroke depression (PSD) of different severity. *p < .01, **p < .001.

Compared with patients in the non‐PSD group, patients with PSD had higher serum levels of HCY (18.90 μmol/L [14.38–23.48 μmol/L] vs. 13.18 μmol/L [10.44–15.72 μmol/L], *p* <.001) (Table 1). Correlation analyses revealed that serum levels of HCY at admission were positively correlated with the HAMD scores 2 weeks after stroke onset in all patients (*r* = 0.532, *p* <.001) (Figure 2) (Table S1). Among the 97 patients in the PSD group, according to the HAMD scores, there were 64 patients of mild depression, 27 patients of moderate depression, and six patients of severe depression (33 patients of moderate‐to‐severe depression). Serum levels of HCY gradually increased with increasing severity of depression as defined by the HAMD‐17 scores (Figure 3). In addition, patients with moderate‐to‐severe PSD had higher HCY levels than patients with mild PSD (22.80 μmol/L [19.65–28.10 μmol/L] vs. 17.04 μmol/L [12.89–20.94 μmol/L], *p* < .01) (Table S2).

Variables with *p* < .05 were included in the multivariable logistic regression model (Table 3). These clinical variables included NIHSS score at admission, serum levels of HCY, quantity of acute infarct, and lesion location. The results indicate that NIHSS scores at admission (OR = 1.288, 95% CI: 1.092–1.520, *p =*.003), serum levels of HCY (OR = 1.169, 95% CI: 1.103–1.239, *p* <.001), and multiple‐site acute infarcts (OR = 2.092, 95% CI: 1.018–4.299, *p =*.045) were independently related to early‐onset PSD. In reference to parietal lobe lesion, there were underlying trends that patients with frontal lobe lesion (*p =*.08) or basal ganglia lesion (*p =*.06) were more likely to be present in the PSD group. But the trends did not reach statistical significance.

**TABLE 3:** Multivariate logistic regression analysis for patients with early‐onset post‐stroke depression (PSD).

	Multivariate Analysis		
Characteristics	OR	95% CI	*p*‐Value
NIHSS score at admission	1.288	1.092–1.520	.003
HCY	1.169	1.103–1.239	<.001
HCY (≥16.98 μmol/L)	10.976	5.585–21.573	<.001
Multiple‐site acute infarcts	2.092	1.018–4.299	.045
Lesion location			
Parietal lobe	1^a^		
Frontal lobe	5.540	0.814–37.684	.080
Temporal lobe	4.936	0.501–48.640	.171
Occipital lobe	2.304	0.142–37.386	.557
Basal ganglia	5.734	0.932–35.266	.060
Thalamus	2.557	0.269–24.333	.414
Brain stem	4.142	0.643–26.679	.135
Cerebellum	3.864	0.531–28.112	.182

Abbreviations: CI, confidence interval; HCY, homocysteine; OR, odds ratio.

^a^ Parietal lobe lesion was as a reference.

**FIGURE 4:**
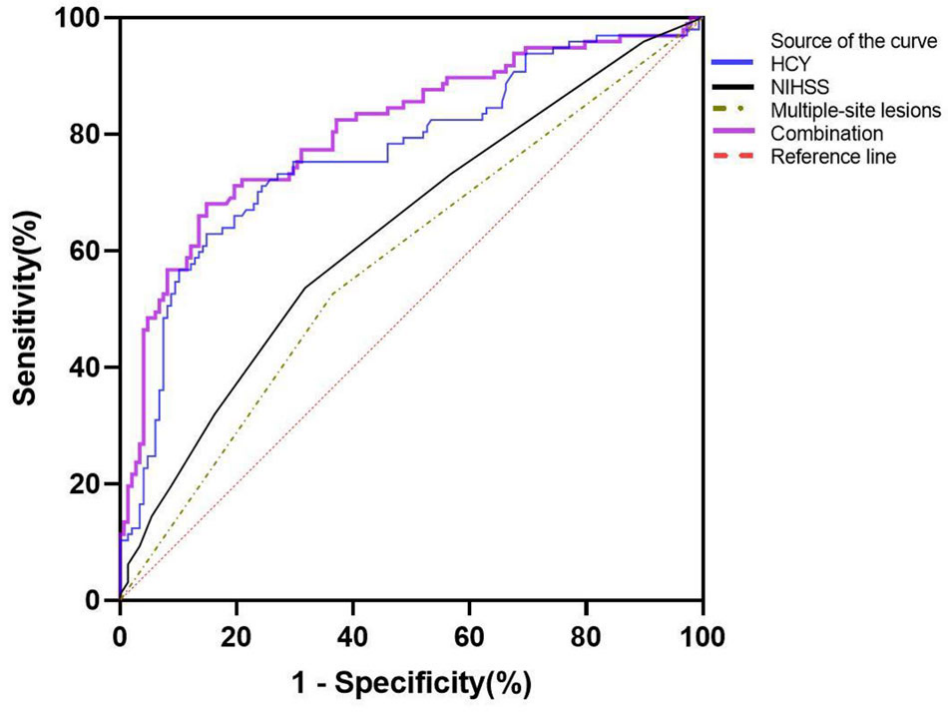
Receiver operator characteristic curve demonstrating sensitivity as a function of 1‐specificity for predicting the early‐onset post‐stroke depression (PSD) based on the combined model and the relative contribution of each factors alone. (area under the curve [AUC], 0.768 [95% CI: 0.704−0.831] for homocysteine [HCY]; AUC, 0.633 [95% CI: 0.562−0.705] for National Institutes of Health Stroke Scale (NIHSS) at admission; AUC, 0.580 [95% CI: 0.507−0.654] for multiple‐site lesions; AUC, 0.807 [95% CI:0.748−0.865] for Combination).

Based on the results of the multivariable logistic regression analysis, all independent prognostic factors for early‐onset PSD were brought into ROC curve analysis (Figure 4). As the optimal cut‐off value, serum levels of HCY≥16.98 μmol/L at admission predicted the early‐onset PSD, with modest sensitivity and high specificity (62.9% and 85.1%, respectively) and area under the curve (AUC)was 0.768 (95% CI: 0.704−0.831, *p* <.001). Serum levels of HCY had a higher prognostic accuracy compared to NIHSS scores at admission (AUC, 0.633 [95% CI: 0.562−0.705], *p* <.001) and multiple‐site lesions (AUC, 0.580 [95% CI: 0.507−0.654], *p =*.033). The combination of HCY, NIHSS scores, multiple‐site lesions, and lesion location revealed a greater prognostic accuracy (AUC, 0.807 [95% CI: 0.748−0.865], *p* <.001) than HCY alone (Table S3). Furthermore, there was a significantly increased risk of early‐onset PSD associated with higher serum levels of HCY ≥ 16.98 μmol/L (OR = 10.976, 95% CI: 5.585–21.573, *p* <.001) after adjustment for above‐recorded variables.

## 4 DISCUSSIONS

With the rapid increase of aging population in China, there is a high overall prevalence, incidence, and mortality rate of stroke and a huge economic and social burden of stroke in China (Tu et al., 2023). PSD is a common complication after stroke and seriously affects the functional rehabilitation and prognosis of patients with stroke, while increasing the mortality rate and family and social burden (Cai et al., 2019; Guo et al., 2022). PSD can occur in different stages after stroke, including the acute stage (within 1 month), the medium stage (1–6 months), and the recovery stage (>6 months). Early‐onset PSD refers to patients exhibiting depression within 2 weeks after acute stroke onset (J. Huang et al., 2019; Lin et al., 2019). Early‐onset PSD exhibits more depression symptoms than late‐onset PSD and is also significantly related to a high risk of unfavorable outcome (mRS score ≥ 3) (Medeiros et al., 2020; Zeng et al., 2021). In our study, the prevalence of early‐onset PSD was 39.6%, with mild‐to‐moderate depression predominating. Furthermore, we found that PSD was associated with stroke severity and activities of daily living. Patients in PSD group had higher NIHSS score and mRS score and lower BI score than patients in non‐PSD group, which indicated that physical impairment and disability were more severe in patients with PSD. NIHSS score at admission was independently associated with early‐onset PSD. This indicated that the severity of stroke was positively related to early‐onset PSD and that the severity of stroke (higher NIHSS score) might be a predictor of early‐onset PSD.

In our study, we found that serum levels of HCY was independently related to early‐onset PSD after adjustment by variables, and compared with patients with lower HCY levels, those with higher HCY levels (≥16.98 μmol/L) had a 10.976‐fold increased risk of early‐onset PSD. The result indicated that high serum levels of HCY in the acute phase of ischemic stroke may be a risk factor for early‐onset PSD. Consistent with our findings, Tang et al. (2016) and J. Zhang et al. (2022) reported that elevated serum levels of HCY at admission were related to depression 2 weeks and 6 months after the stroke onset. Through meta‐analysis, Y. Chen et al. (2022) also found that serum levels of HCY could be used as a biomarker for the prediction for the risk of early‐onset PSD. This HCY biomarker is easily detected in clinical practice and has good predictive accuracy (76.8%) in our study. This study also suggested that the combination of HCY, stroke severity, and lesion localization could provide more predictive information for early‐onset PSD.

HCY is an essential intermediate product during the metabolism of methionine to cysteine. The transformation process of HCY in vivo is aided through several enzymes and three vitamins, folic acid, B12, and B6. Deficiency of folic acid, vitamins B6, and B12 can lead to HCY transformation and clearance disorders, leading to serum levels of HCY elevation (Hankey, 2018; Kaye et al., 2020; Poddar, 2021). It is believed that high HCY is related to the occurrence and development of cardiovascular and cerebrovascular diseases. Many studies have shown that hyperhomocysteinemia is an independent risk factor for cerebral infarction and atherosclerosis (Ganguly & Alam, 2015; Poddar, 2021; T. Zhang et al., 2020). Vitamin B supplementation therapy including vitamin B6, B12, and folic acid, especially in stroke patients, can effectively reduce HCY levels, thereby reducing the risk and recurrence of ischemic stroke (X. Huang et al., 2017; Kataria et al., 2021; Li et al., 2016; Yuan et al., 2021). The mechanisms underlying the relationship of elevated HCY levels and PSD have not been fully elucidated. There are several mechanisms that might explain the role of HCY in PSD. First, elevated HCY levels can cause a decrease in the synthesis of S‐adenosylmethionine (SAM) in the methionine cycle, which is a methyl donor for various methylation reactions in the body. SAM is also involved in the synthesis and metabolism of neurotransmitters such as 5‐hydroxytryptamine, norepinephrine, and dopamine. The decrease of SAM results in the reduced synthesis and metabolic disorder of the three neurotransmitters related to emotions, leading to a depressive state (Bhatia & Singh, 2015). Second, hyperhomocysteinemia exerts toxicity to neuronal and endothelial cells. High HCY is also oxidized to excitatory amino acid (EAA) neurotransmitters, such as homocysteic acid (HCA) and cysteine sulfinic acid (CSA). HCA and CSA lead to activation of N‐methyl‐Daspartate (NMDA) glutamate receptor. Activation of NMDA receptor promotes calcium influx into neuronal cells, further leading to apoptosis or changes in the ability of neurons to transmit signals (Bhatia & Singh, 2015; Poddar et al., 2017). In a rat ischemic brain model, elevated HCY levels caused mitochondrial dysfunction, increased reactive oxygen species productions, an induced mitochondrial‐dependent apoptotic pathways causing cortical and hippocampal neuronal cell injury in the rat ischemic brain (S. Chen et al., 2017). Apoptosis of hippocampal neurons promotes the occurrence and development of depression. Through regulating the ionotropic glutamate receptors, HCY exerts neurotoxic effects on hippocampal neuronal cell and induces hippocampal neuronal apoptosis (Kang et al., 2020). Third, elevated HCY levels result in vascular endothelial dysfunction. Through inhibiting the catalytic activity of nitric oxide synthase, HCY was correlated with reduced nitric oxide (NO) production, which was attributed to a key vasodilator factor in endothelium. Meanwhile, by activating nuclear factor kappa B, HCY participates in the production of pro‐inflammatory cytokines, such as macrophage chemoattractant peptide‐1 (MCP‐1) and interleukin‐8. The increase of these proinflammatory cytokines, damage and apoptosis of vascular endothelial cells, and destruction of the blood brain barrier are all associated with the occurrence and development of depression (Esse et al., 2019; Hayley et al., 2021).

Using visual analysis of CT or MRI images, previous clinical studies have shown that anatomic allocation of stroke lesions, laterality and quantity of lesions are associated with the prevalence and severity of PSD. Some specific brain areas, such as frontal lobe, basal ganglia, and limbic system, which are involved in regulating emotions, are damaged after stroke onset and result in PSD. Robinson and Jorge (2016) support these opinions that the onset and severity of PSD are significantly related to left frontal hemisphere lesions and ischemic lesions close to the frontal pole. Furthermore, there is an association between PSD and left frontal or left basal ganglia lesions within 2 months after acute stroke. Through meta‐analysis, Douven et al. (2017) found that patients with frontal or basal ganglia lesions had an obviously higher incidence of PSD in the post‐acute phase (15 days to 6 months after stroke onset). Nevertheless, left hemispheric lesions were more likely to lead to PSD in the acute phase (<15 days from stroke onset). In our study, we found that stroke lesion laterality was not related to PSD. There were underlying trends that patients with frontal lobe lesion or basal ganglia lesion were more likely to occur in the PSD group. Although the trends did not reach statistical significance, the results indicated that the frontal lobe may play a more important role in regulating emotions than other brain regions, which is consistent with a previous study's results (Medeiros et al., 2020). In recent years, based on the development of functional MRI such as diffusion tensor imaging (DTI), it has been proposed that stroke lesions affect specific brain regions within fronto‐striatal brain networks, leading to the onset of PSD. Structural damage of white matter fiber tracts connecting frontal and subcortical brain regions, particularly fronto‐striato‐thalamic pathways, is associated with PSD. Structural alterations of anterior interhemispheric connections also relate to emotion regulation of patients with PSD (G. Chen et al., 2016; Nickel & Thomalla, 2017). The limbic‐cortical‐striatal‐pallidal‐thalamic (LCSPT) circuit plays a significant role in the pathogenesis of depression. Therefore, microstructural alterations in the circuit may induce abnormal regional junctions, which are related to emotional disorders of patients with PSD. Meanwhile, Xu et al. (2019) consider that the degree of damage to white matter fiber tracts might be related to the symptoms and severity of PSD. These findings might provide a link between structural brain lesions and PSD. In our study, multiple‐site acute infarcts are independently related to early‐onset PSD. PSD is more prevalent in patients with multiple stroke lesions. Our finding is consistent with the results by Jiang et al. (2014). The result indicated that multifocal lesions damage brain structures related to emotional regulation, including the brain network structures associated with the prefrontal lobe, basal ganglia, and thalamus, as well as the white matter fiber tracts of LCSPT circuit, leading to the occurrence of early onset PSD. Meanwhile, we found that the combination of HCY, NIHSS scores, multiple‐site lesions, and lesion location revealed a greater prognostic accuracy (80.7%) for the risk of early‐onset PSD. These results demonstrated the superimposed effect of these risk factors on the predictive value and reflected more overall pathogenesis of early onset PSD.

There are several limitations to this study. First, this was a single‐center study, and the sample size was relatively small. Sampling and selection biases may be inevitable; thus, further clinical studies with a large sample size in multiple centers are warranted. Second, we excluded patients with severe aphasia or unclear consciousness or dementia during hospitalization from the study. However, these patients may have experienced a more severe stroke and might have a more severe depression. Third, some risk factors that may influence depressive episodes including social support, educational background, and increased life stress were not able to be examined. Fourth, we only observed PSD at 2 weeks after stroke onset. Conclusions from short‐term observational studies might not be comprehensive enough. The secular variation of HCY levels with stroke course and its relationship with PSD need further study. The effect of early treatment including IV‐tPA thrombolysis or endovascular thrombectomy on HCY also needs further research. Further large‐scale clinical cohort studies with a longer term intervention and follow‐up are needed to fully evaluate how HCY levels influence the occurrence of PSD.

## 5 CONCLUSION

Despite these limitations, our study indicated that higher NIHSS score, elevated serum levels of HCY, and multiple‐site stroke lesions may be independent risk factors of early‐onset PSD. Additionally, the combination of HCY, NIHSS scores, multiple‐site stroke lesions, and lesion location may provide greater predictive value than HCY alone for early‐onset PSD. Our study suggested that these factors might participate in the pathophysiology alterations of depression symptoms in patients with stroke. Early intervention for elevated serum levels of HCY may be a potential target for intervention and prevention of PSD.

## AUTHOR CONTRIBUTIONS

Hongxu Zhou, Chenlong Wang, and Wei Wang were responsible for the conception and design of the study. Hongxu Zhou, Chenlong Wang, Wei Wang, Ni Huang, Hongyan Li, and Qun Hu were in charge of recruiting and evaluating patients and data collection. H.Z, Wei Wang, and Qun Hu were in involved in analysis and interpretation of data. Hongxu Zhou was involved in manuscript drafting. Chenlong Wang and Yining Huang were responsible for the critical revision of the manuscript. All authors contributed to the article and approved the submitted version.

## CONFLICT OF INTEREST STATEMENT

The authors declare no conflict of interest.

### PEER REVIEW

The peer review history for this article is available at https://publons.com/publon/10.1002/brb3.3210.

## Supporting information

Table_1_SuppInfo The correlation between the HAMD score and characteristics
Table_2_SuppInfo Comparison of serum HCY level among PSD of different severity
Table_3_SuppInfo Area under the curve of ROC analysis

## Data Availability

The data that support the findings of this study are available from the corresponding author upon reasonable request.
