# Supplementary material for: Lesion location and serum levels of homocysteine are associated with early‐onset post‐stroke depression in acute ischemic stroke
Source: Brain Behav. 2023 Aug 16;13(10):e3210. doi: 10.1002/brb3.3210 (PMC10570478; doi:10.1002/brb3.3210)
Supplement: Supplementary file 1 — Table_1_SuppInfo The correlation between the HAMD score and characteristics Table_2_SuppInfo Comparison of serum HCY level among PSD of different severity Table_3_SuppInfo Area under the curve of ROC analysis [file BRB3-13-e3210-s001.docx]

Table_1_SuppInfo The correlation between the HAMD score and characteristics

| HAMD score | *r*_s_ | *P* |
| --- | --- | --- |
| Age | 0.019 | 0.762 |
| NIHSS score | 0.314 | ＜0.001 |
| BI score | -0.282 | ＜0.001 |
| mRS score | 0.411 | ＜0.001 |
| Systolic blood pressure | 0.132 | 0.039 |
| LDL-C | 0.093 | 0.148 |
| FBG | -0.071 | 0.267 |
| HbA1c | -0.037 | 0.559 |
| Uric acid | 0.022 | 0.727 |
| HCY | 0.532 | ＜0.001 |
| CRP | 0.154 | 0.016 |

*r*_s_: correlation coefficient(Spearman's Rank correlation analysis)

Table_2_SuppInfo Comparison of serum HCY level among PSD of different severity

| Group | N | HAMD, median(IQR) | HCY, median(IQR), μmol/L | Z value | *P* |
| --- | --- | --- | --- | --- | --- |
| Non-PSD | 148 | 5(4-6) | 13.18(10.44-15.72) |  |  |
| Mild PSD | 64 | 11(9-14) | 17.04(12.89-20.94) | 4.556^a^ | ＜0.001^a^ |
| Moderate- severe PSD | 33 | 19(18-21) | 22.80(19.65-28.10) | 7.259^a^  3.340^b^ | ＜0.001^a^  ＜0.003^b^ |

a: compared with non-PSD; b: compared with mild PSD

Table_3_SuppInfo Area under the curve of ROC analysis

| Variables | Area | 95% Confidence Interval | *P* |
| --- | --- | --- | --- |
| NIHSS score | 0.633 | 0.562-0.705 | ＜0.001 |
| Multiple-site lesions | 0.580 | 0.507-0.654 | 0.033 |
| HCY | 0.768 | 0.704-0.831 | ＜0.001 |
| Combination^*^ | 0.807 | 0.748-0.865 | ＜0.001 |

ROC: receiver-operating characteristic curves

*: the combination of HCY, NIHSS score, multiple-site lesions and lesion location
